# The role of rice fields, fish ponds and water canals for transmission of fish-borne zoonotic trematodes in aquaculture ponds in Nam Dinh Province, Vietnam

**DOI:** 10.1186/s13071-015-1237-z

**Published:** 2015-12-08

**Authors:** Henry Madsen, Bui Thi Dung, Dang Tat The, Nguyen Khue Viet, Anders Dalsgaard, Phan Thi Van

**Affiliations:** Department of Veterinary Disease Biology, Faculty of Health and Medical Sciences, University of Copenhagen, Frederiksberg, Denmark; Institute of Ecology and Biological Resources, Vietnam Academy of Science and Technology, Hanoi, Vietnam; Research Institute for Aquaculture No. 1, Tu Son, Vietnam

**Keywords:** Haplorchis, Clonorchis, Melanoides, Bithynia, aquaculture

## Abstract

**Background:**

Fish-borne zoonotic trematodes (FZT), such as *Clonorchis sinensis*, *Opistorchis viverini* (Opisthorchiidae) and intestinal trematodes of the family Heterophyidae, constitute a public health hazard in Vietnam and infections with these trematodes has been linked to consumption of raw or undercooked fish from aquaculture. The FZT transmission pathways, however, are more complicated than just the presence of intermediate snail hosts in aquaculture ponds as ponds may exchange water with surrounding habitats such as rice fields and irrigation canals and thereby these surrounding habitats may be a source of snails and cercariae and contribute to FZT infection in cultured fish.

**Methods:**

This is a longitudinal descriptive study on selected farms (*n* = 30) in Nam Dinh Province which is endemic for FZT. At each farm, we sampled one pond, a small irrigation canal used to supply the pond with water, and a nearby rice field. At each of these three sites, we estimated the density of the FZT intermediate snail hosts and determined their trematode infection status. Comparative analysis was performed for the prevalence and density of FZT infections in fish and snails.

**Results:**

Species of the Thiaridae, and most notably *Melanoides tuberculata*, the most important host species for FZT belonging to the Heterophyidae, were particularly abundant in ponds and small canals, i.e. *M. tuberculata* was found in 27 ponds and 13 small canals. *Bithynia fuchsiana*, a potential host for both Heterophyidae and Opisthorchiidae, was rarely found in fish ponds but common in rice fields. A total of 12 types of cercariae were found in the snails and pleurolophocercous cercariae, primarily FZT, constituted about 40 % of all cercarial infections. The fish species cultured were mainly carp species and *Haplorchis pumilio* was the dominating trematode species infecting fish. *Clonorchis* spp. were not recorded in any of the ponds. FZT transmission to fish was intense during the summer period (May-June to November) but less intense during the winter months (December-January) partly because cercarial emergence ceases due to the low temperature.

**Conclusion:**

Our findings highlight the complexity of FZT transmission within aquaculture farm settings and suggest that efforts to control these infections must take a holistic approach using interventions against all stages of the transmission cycle.

## Background

Fish-borne zoonotic trematodes (FZT), such as the liver flukes *Clonorchis sinensis* and *Opisthorchis viverini* (Opisthorchiidae) as well as intestinal trematodes of the family Heterophyidae, constitute a public health hazard in Vietnam and elsewhere in Asia [1–7]. Infections with these trematodes have been linked to consumption of raw or undercooked fish from aquaculture, although the relative importance of cultured *vs* wild-caught fish as sources of human infection remains uncertain [8–11]. The FZT use certain freshwater snail species as first intermediate hosts [12] and presence of these gastropods in aquaculture ponds is an essential risk factor when fish are infected [13]. The transmission pathways and risks, however, are more complicated than just the presence of snails in aquaculture ponds, e.g. Clausen *et al.* [13] found high prevalence of FZT infection among fish from nursery ponds where snail density was low or even nil. The FZT transmission is further complicated by the fact that domestic animals and possibly fish-eating birds serve as final hosts as well [14].

Fish culture in Vietnam ranges from small family-based integrated fish ponds, the so-called VAC (Vuon: garden Ao ca: pond; Chuong: pigsty) ponds, to large scale commercial pond or cage mono culture systems, such as *Pangasius hypophthalmus* (striped catfish) culture in the Mekong River Delta, and for each production system there are different risk factors for infections with FZT [15–19]. The intermediate snail hosts for the intestinal trematodes (Heterophyidae), which are highly prevalent in aquaculture systems in Vietnam [16, 19], are primarily species belonging to the Thiaridae, especially *Melanoides tuberculata*, but other species, i.e. *Thiara scabra*, *Tarebia granifera*, and *Sermyla riquetti* are also important hosts [15]. In addition, species of the Bithyniidae are also hosts for intestinal trematodes and the most important intermediate hosts for liver trematodes (Opisthorchiidae) [15].

There is a considerable production of cercariae in snail hosts in various habitats surrounding fish ponds such as rice fields and small water canals [15] and since ponds may exchange water with these habitats, there is a risk that metacercariae found in fish may be of allochthonous origin. Other sources and means of introduction of cercariae or snails could be the feeding of snails to ducks or fish, e.g. to the black carp (*Mylopharyngodon piceus*) commonly stocked in aquaculture ponds, but also feeding aquatic vegetation to grass carps [16, 20, 21]. Thus different farm management practices together with environmental factors such as temperature and rainfall are important determinants of FZT transmission and risk of infection [5, 8].

This longitudinal study attempted to determine whether snail density and prevalence of FZT infected snails in rice fields, small water canals and aquaculture ponds were associated with FZT prevalence or intensity of infection in fish from grow-out ponds in Nam Dinh province, an FZT endemic area in northern Vietnam.

## Methods

### Study area

**Fig. 1:**
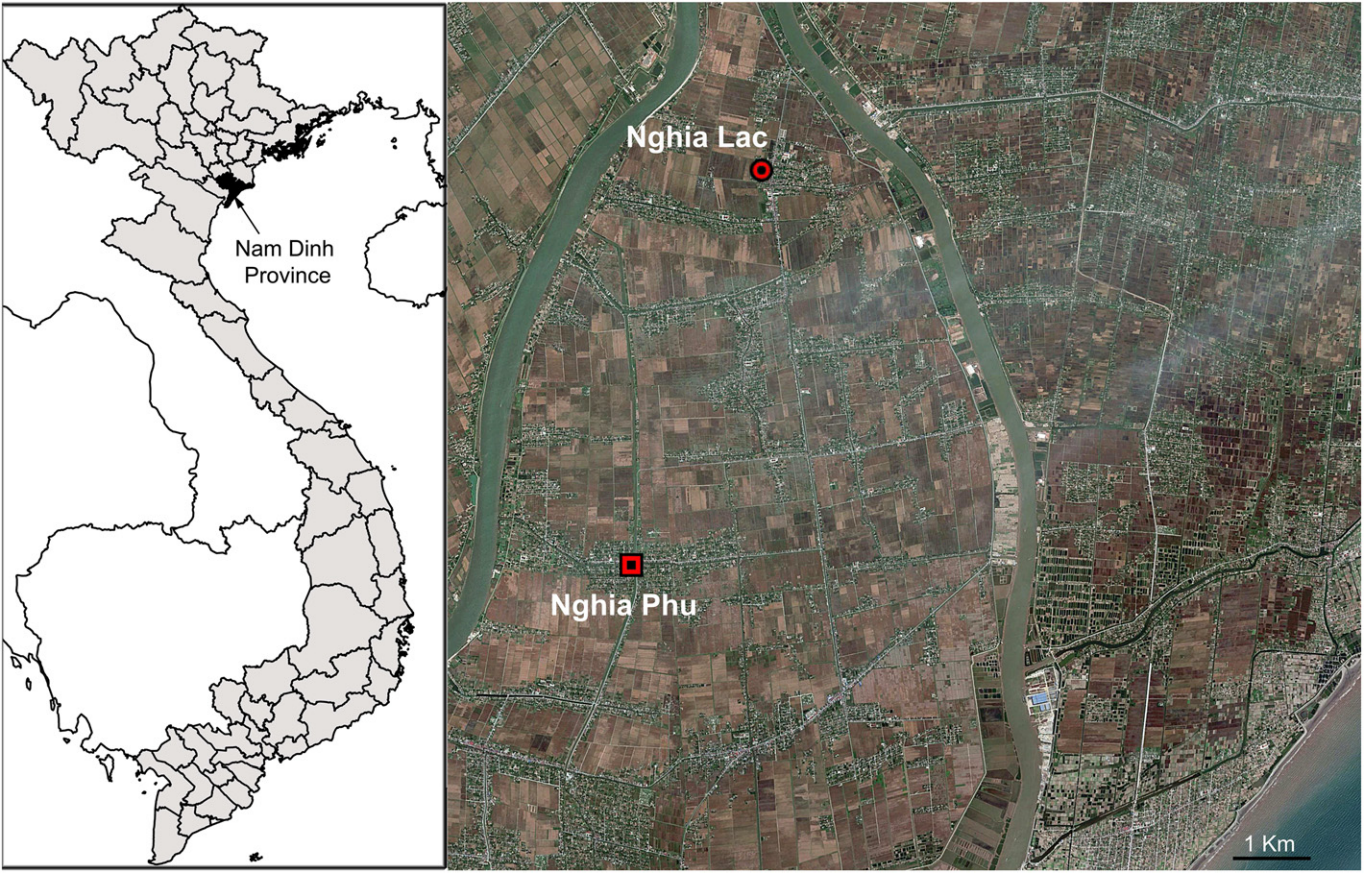
Map showing location of the study area. Map copied from Google Earth

The study was conducted from June 2005 to July 2006 in Nghia Lac and Nghia Phu communes in the Nghia Hung District in Nam Dinh province, which is located in the Red River Delta in Northern Vietnam [15]. Nam Dinh province (Fig. 1) is a coastal province located at the southeast of the Red River delta (19°55′–20°16′N, 106°00′–106°33′E). The main stocking season for juvenile fish in grow out ponds is late spring (April-May) and beginning of summer; during other times of the year, juveniles are stocked at convenience by farmers [16].

For snail sampling, we selected 30 fish farms (study unit), 15 from Nghia Lac and 15 from Nghia Phu commune (Fig. 1). Distance between any pair of two farms ranged from about 100 m to maximum 6–7 km. In each commune, two of the 15 farms were nursery farms producing juveniles while the remaining 26 farms were grow-out farms. Each selected farm had a fish pond, a nearby small canal from where the pond was supplied with water and a closely located rice field (not necessarily directly connected to the pond). Distance from the canal to the pond ranged from a few metres to 20 m and the distance from the pond to the rice field ranged from about 10 m to 50 m. Twenty-five of these grow-out farms were also sampled by Phan *et al.* [16] to identify the main risk factors associated with FZT transmission. The sample size was mainly determined by the number of grow-out ponds sampled previously [16] and the capacity of the laboratory to process and analyse snail samples. Fish were sampled every second month while snail sampling was less regular with more frequent sampling during the winter period, because we wanted to know how the agricultural activities and low temperature during the winter months affected FZT transmission.

### Snail sampling and identification

Sampling sites in small canals were defined as a 50 m stretch around the water intake for the aquaculture pond and in rice fields as a 50 m stretch close to the pond. In ponds the entire periphery if accessible was considered a sampling site. Thus for each farm 3 samples (1 in each habitat) were taken at each time point. Snail sampling was conducted by the same two people at all sites during the morning hours for 30 min per site using a scooping method and/or hand-picking (only in rice fields). Snails were transferred to plastic containers and transported alive to a temporary field laboratory where they were identified to species according to keys [22, 23]. Snails were then microscopically examined for trematode infection status (not infected, infected), using emergence or dissection [15], and cercariae were identified only to major group [24]. For emergence, snails were exposed to electric light for 3–4 hours and checked for cercariae emergence. Snails were left in the containers overnight and rechecked the following morning.

### Fish metacercariae analysis

The main species stocked were grass carp (*Ctenopharyngodon idellus*), silver carp (*Hypophthalmichthys molitrix*), indian carp (*Labeo rohita*), and mrigal carp (*Cirrhinus mrigala*) [16]. Other species sampled included common carp (*Cyprinus carpio*), red-eyed carp (*Squaliobarbus curriculus*), crucian carp (*Carrasius auratus*), mud carp (*Cirrhinus mlitorella*), pacu (*Piaractus brachypomum*), climbing perch (*Anabas testudineus*), tilapia (*Oreochromis niloticus*) and a few specimens of other species. The stocked juveniles mainly originated from local nurseries.

Fish were sampled using a cast net thrown 5 times as described by Phan *et al.* [16]. All fish captured were pooled in a container and 10 fish were randomly picked for FZT examination; if, however, less than 15 fish were caught, all were selected and analysed. The length and weight of each fish were recorded before being processed and digested in 1 % pepsin for the release of metacercariae [16]. Small fish (<200 g) were ground and digested whole. For larger fish (>200 g), the whole fish was ground using an electrical grinder, mixed well and a 50 g subsample was obtained and digested for recovery of metacercariae. Metacercariae were identified [16] and counted by species and the density determined (no. of metacercariae/g tissue).

### Ethics statement

The study was done following the European Convention for the “Protection of Vertebrate Animals used for Experimental and Other Scientific Purposes”. Animals were handled with respect according to the study protocol which was approved by the Aquatic Animal Scientific Committee of Research Institute for Aquaculture No.1, under The Ministry of Agriculture and Rural Development, Vietnam.

### Statistical analysis

Shannon’s diversity index [25] was used to compare diversity of snails or trematodes (as cercariae morphotypes) among habitats (pond, small canal and rice field). Snail or cercariae counts were aggregated by sampling site and the Shannon diversity index calculated for each site from where these calculations were possible, i.e. sites without snails or cercariae respectively were excluded. Similarly, the diversity index for cercariae types was calculated by snail species by aggregating all cercariae infections found in a given snail species.

Metacercariae counts in fish were analysed using negative binomial regression [26] with weight of tissue examined as an offset. Predictors of metacercariae density in fish were tested individually together with the effect of sampling date and then jointly in one analysis, where insignificant predictors were removed and for categorical predictors, categories that did not differ significantly were combined. In all analyses, household was considered as a cluster. In the figures, snail density was expressed as number collected per 30 min search and metacercariae density as number per gram fish tissue. Snail or metacercariae counts were log (× + 1)-transformed; the means of these transformed counts and their confidence intervals were then back-transformed to the original scale.

## Results

### Snail fauna

Approximately, 25,000 snails representing a total of 15 species were found (Table 1). About half of all snails collected belonged to the Thiaridae, with *Melanoides tuberculata* being the most common and abundant species (Table 1). Thiarid snails were particularly abundant in ponds and small canals (Table 1). *Thiara scabra* and *Tarebia granifera* were not recorded from rice fields, while both *M. tuberculata* and *Sermyla riquetti* each constituted about 10 % of all snails found in rice fields (Table 1). *Bithynia fuchsiana* was uncommon in fish ponds but common in rice fields, while *Stenothyra messageri* was primarily found in small canals (Table 1). *Angulyagra polyzonata* was common in fish ponds (29/30 ponds) and in small canals (15/30 canals). The most common snail species in rice fields were *Lymnaea* spp. (primarily *L. viridis*), *B. fuchsiana* and *Gyraulus convexiusculus* (Table 1). Snails of at least one species were found in 22/30 sites in small canals, 19/30 rice fields and in 29/30 fish ponds. Diversity of snails was slightly higher in small canals (Shannon’s diversity index 0.892 ± 0.195, *n* = 22) and rice fields (Shannon’s diversity index 0.887 ± 0.213; *n* = 19) than in fish ponds (Shannon’s diversity index 0.628 ± 0.103, *n* = 29).

**Table 1:** Snail fauna and distribution in small water canals, rice fields and fish ponds each represented by 30 sites located in two communes, Nhgia Lac and Nhgia Phu, in Nam Dinh province, northern Vietnam

	Small canals (*n* = 30)			Rice fields (*n* = 30)			Fish ponds (*n* = 30)		
Snail species	No. of sites	No. of snails	%	No. of sites	No. of snails	%	No. of sites	No. of snails	%
Thiaridae									
*Thiara scabra*	6	1641	19.1	0	0	0.0	13	1214	10.4
*Tarebia granifera*	0	0	0.0	0	0	0.0	3	601	5.2
*Melanoides tuberculata*	13	2697	31.3	10	480	10.1	27	4637	39.9
*Sermyla riquetii*	2	346	4.0	1	465	9.7	5	1892	16.3
Bithynidae									
*Bithynia fuchsiana*	5	167	1.9	17	984	20.6	5	23	0.2
Stenothyridae									
*Stenothyra messageri*	11	1342	15.6	7	236	4.9	1	5	0.0
Other									
*Neritina violacea*	1	6	0.1	0	0	0.0	1	1	0.0
*Angulyagra polyzonata*	15	927	10.8	4	33	0.7	29	3202	27.5
*Idiopoma umbilicata*	1	6	0.1	1	4	0.1	0	0	0.0
*Pila polita*	0	0	0.0	0	0	0.0	2	2	0.0
*Pomacea canaliculata*	8	48	0.6	2	2	0.1	5	16	0.1
*Pomacea insularum*	9	125	1.5	9	200	4.2	7	13	0.1
*Polypylis hemisphaerula*	8	400	4.6	6	26	0.5	1	16	0.1
*Gyraulus convexiusculus*	12	864	10.0	14	461	9.7	1	5	0.0
*Lymnaea* spp.^a^	4	34	0.4	9	1878	39.3	4	6	0.1

Number of sites where a species was found, the total number of snails collected and the relative abundance (in %), i.e. the contribution of each species to the total number of snails collected, are reported

^a^A few *L. swinhoei* but mainly *L. viridis*

### Cercariae types

A total of about 1900 snails were found infected with trematodes and 12 cercariae types were found in the water canals, rice fields and fish ponds (Table 2). Pleurolophocercous cercariae constituted about 40 % of all cercariae infections, echinostomes about 28 % and xiphidiocercariae about 25 % (Table 2). Echinostome and xiphidiocercariae were found in several snail species (Table 2). *Melanoides tuberculata* was the most common host for pleurolophocercous cercariae in all three habitats with the prevalence of infection being highest in small canals (Table 3). Pleurolophocercous cercariae were also found in the other thiarid species and in *Stenothyra messageri*, but not in *Bithynia fuchsiana*. Thiarid snails were also hosts for the echinostomes and xiphidiocercariae and so was *Bithynia fuchsiana*, especially in rice fields (Table 3). Diversity of cercariae types by habitat did not differ significantly, i.e. Shannon’s diversity index 1.030 ± 0.333, 0.949 ± 0.390 and 1.109 ± 0.191 in small canals (*n* = 10), rice fields (*n* = 7) and ponds (*n* = 19), respectively. Diversity of cercariae morphotypes was highest in *M. tuberculata* (Shannon’s diversity index 1.642), followed by *Tarebia granifera* (Shannon’s diversity index 1.441), *Sermyla riqueti* (Shannon’s diversity index 1.187) while Shannon’s diversity index for the remaining species had scores below 1.000.

**Table 2:** Relative abundance (% of all infections in snails) of cercariae morphotypes found in snails collected in small canals, rice fields and fish ponds in in two communes, Nhgia Lac and Nhgia Phu, in Nam Dinh province, northern Vietnam

Morphotypes of cercariae	No. of infected snails	Relative abundance (%)	No. of infected snail species	No. of sites where found		
				Small canals (*n* = 30)	Rice fields (*n* = 30)	Ponds (*n* = 30)
Echinostome cercariae						
Type 1	495	26.3	7	9	6	17
Type 2	27	1.4	2	2	1	1
Pleurolophocercous cercariae						
Type 1	360	19.2	4	8	4	17
Type 2	379	20.2	4	9	5	16
Type 3	19	1.1	1	1	1	2
Type 4	18	1.0	4	3	2	2
Other						
Amphistome cercariae	2	0.1	2	1	1	0
Furcocercariae	6	0.3	3	0	2	2
Gymnocephalous cercariae	34	1.8	3	1	0	4
Monostome cercariae	69	3.7	3	3	0	9
Opthalmoxiphidiocercariae	2	0.1	1	1	0	0
Xiphidiocercariae	469	25.0	6	10	10	16

**Table 3:** Number of snails examined for trematode infections (in bold) and number of snails infected with different cercariae morphotypes in two communes, Nhgia Lac and Nhgia Phu, in Nam Dinh province, northern Vietnam

	Small canals		Rice fields		Fish ponds		All habitats	
Snail taxon and cercariae type	No. of snails	*P* (%)	No. of snails	*P* (%)	No. of snails	*P* (%)	No. of snails	*P* (%)
*Melanoides tuberculata*	2618		480		4601		7699	
Parapleurolophocercous	348	13.3	44	9.2	292	6.3	684	8.9
Echinostome	220	8.4	28	5.8	121	2.6	369	4.8
Xiphidiocercariae	110	4.2	26	5.4	189	4.1	325	4.2
Other	12	0.5	0	0.0	67	1.5	79	1.0
Other thiarids	1986		465		3705		6156	
Parapleurolophocercous	45	2.3	0	0.0	22	0.6	67	1.1
Echinostome	73	3.7	0	0.0	36	1.0	109	1.8
Xiphidiocercariae	14	0.7	0	0.0	57	1.5	71	1.2
Other	0	0.0	0	0.0	26	0.7	26	0.4
*Bithynia fuchsiana*	155		861		23		1039	
Parapleurolophocercous	0	0.0	0	0.0	0	0.0	0	0.0
Echinostome	0	0.0	9	1.0	1	4.3	10	1.0
Xiphidiocercariae	14	9.0	50	5.8	3	13.0	67	6.4
Other	0	0.0	2	0.2	0	0.0	2	0.2
*Stenothyra messageri*	1339		236		5		1580	
Parapleurolophocercous	22	1.6	2	0.8	0	0.0	24	1.5
Echinostome	0	0.0	0	0.0	0	0.0	0	0.0
Xiphidiocercariae	5	0.4	0	0.0	0	0.0	5	0.3
Other	1	0.1	0	0.0	0	0.0	1	0.1
Other species	1512		2231		1365		5108	
Echinostome	5	0.3	26	1.2	3	0.2	34	0.7
Xiphidiocercariae	0	0.0	1	0.0	0	0.0	1	0.0
Other	2	0.1	3	0.1	0	0.0	5	0.1

Overall prevalence of infection (P in %) is also given

Initially, both cercarial emergence and snail dissection were used for detection of cercariae, but during the winter months, especially during the sampling in January, the snails with patent infections did not show cercariae emergence. Therefore, only dissection was done in January. Comparing the two methods for cercariae detection when adjusting for sampling time when both methods were used showed that 33 % (p < 0.01) more snail infections were detected *via* dissection than by the cercarial emergence method.

### Metacercariae in fish

A total of more than 200,000 metacercariae was collected and identified (Table 4). *Clonorchis sinensis* was not recorded from any of the ponds while *Haplorchis pumilio* was the dominating species (Table 4). About 20 % of the metacercariae could not be identified. A total of 1226 fish, mainly carp species, was examined (Table 5). Grass carp had the highest metacercariae densities (metacercariae/g fish weight) and the highest prevalence of metacercariae infection (Table 5).

**Table 4:** Relative abundance (% of all metacercariae recorded) of the metacercariae in fish from aquaculture ponds in two communes, Nhgia Lac and Nhgia Phu, in Nam Dinh province, northern Vietnam

Species	No. recorded	Relative abundance (%)	No. of ponds
*Clonorchis sinensis*	0	0	0
*Haplorchis pumilio*	160,222	77.3	25
*Haplorchis taichui*	261	0.1	8
*Haplorchis yokogawi*	7	0	4
*Centrocestus formosanus*	114	0.3	18
*Exorchis* sp.^a^	21	0	4
*Procerovum* sp.	4147	2.0	13
Other species	94	0.1	7
Not identified	42,147	20.3	22

^a^not zoonotic

**Table 5:** Relative abundance of metacercariae in 25 ponds with matching snail data (fish species represented by less than 10 specimens were grouped together as “Other”)

Fish species	No. of fish	No. of ponds	Mean metacer-cariae density^a^	Maximum density in 1 fish	Weight range (g) of fish	Prevalence of FZT (%)	Count ratio	Odds ratio
Grass carp (*Ctenopharyngodon idellus*)	126	20	9.23	177.7	60–896	90.5	1.00	1.00
Indian carp (*Labeo rohita*)	416	25	0.23	7.4	10–720	70.2	0.02***	0.2***
Mrigal (*Cirrhinus mrigala*)	153	19	0.38	9.7	30–350	79.1	0.04***	0.38*
Pacu (*Piaractus brachypomum*)	31	4	0.78	5.05	20–100	90.3	0.11***	0.87
Red-eyed carp (*Squaliobarbus curriculus*)	39	8	0.24	2.19	80–210	76.9	0.03***	0.32
Silver carp (*Hypophthalmichthys molitrix*)	382	23	4.75	216	20–430	86.9	0.46*	0.59
Crucian carp (*Carrasius auratus*)	66	18	1.09	9.2	18–170	77.3	0.11***	0.27*
Tilapia (*Oreochromis niloticus*)	25	7	0.59	13.8	19–210	32.0	0.06**	0.04***
Other	28	12	4.29	64	12–420	75.0	0.42	0.31

Based on all fish collected in 25 fish ponds in two communes, Nhgia Lac and Nhgia Phu, in Nam Dinh province, northern Vietnam over the entire study period

^a^mean no of metacercariae per g fish; *p < 0.05; **p < 0.01; ***p < 0.001

### Seasonal variation in prevalence and density of FZT infection in fish

**Fig. 2:**
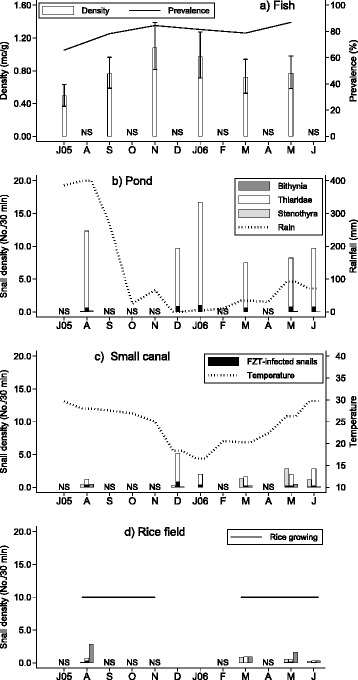
Prevalence and density of FZT infection in fish (a); snail density for three groups of host species (Thiaridae, *Bithynia* and *Stenothyra*) in ponds (b), small canals (c) and rice fields (d) together with rainfall and air temperature from July 2005 to June 2006 in two communes in Nam Dinh Province. The symbols for groups of snail hosts are the same in subfigures (b-d) and the black columns indicate density of FZT infected snails. NS = not sampled

The first sampling of fish was carried out in July and at that time prevalence of FZT infection was already high, i.e. 78.8 % in the *Ctenopharyngodon idellus* (*n* = 33), 83.6 % in the *Hypophthalmichthys molitrix* (*n* = 61), 50.6 % in *Labeo rohita* (*n* = 81) and 69.8 % in *Cirrhinus mrigala* (*n* = 43) with other fish species represented by relatively small numbers. Mean metacercariae densities in July were 3.97, 2.85, 0.12 and 0.06 metacercariae/g for the four fish species, respectively. Both trematode prevalence and density of metacercariae infection increased until November (Fig. 2). In order to analyse the data with fewer covariate patterns, fish species was recoded as *C. idellus, L. rohita*, *C. mrigala*, *H. molitrix* and all other fish species were combined into one group. In a model with these fish species categories, the count ratios between a sampling time and the July sampling were 1.71 (95 % CL: 0.93–3.13; *p* > 0.05), 3.45 (95 % CL: 2.27–5.24; *p* < 0.001), 2.51 (95 % CL: 1.24–5.07; *p* < 0.05), 2.34 (95 % CL: 0.96–5.71; *p* > 0.05) and 1.98 (95 % CL: 1.03–3.83; *p* < 0.05) for the samples of September, November, January, March and May, respectively. In the same model *L. rohita*, *C. mrigala*, *H. molitrix* and other fish species had metacercariae counts of 0.23 (95 % CL: 0.01–0.05; *p* < 0.001), 0.04 (95 %: 0.02–0.08; *p* < 0.001), 0.45 (95 % CL: 0.24–0.86; *p* < 0.05) and 0.13 (95 % CL: 0.05–0.31; *p* < 0.001) of that of *C. idellus,* respectively.

#### The summer period (June/July to November)

Transmission appeared to be intense during the summer period as both the prevalence and intensity of infections in fish increased up to November (Fig. 2a). During this period air temperature was high (monthly mean air temperature range from 29.5 °C in June to 25.0 °C in November) as was rainfall with a total of about 400 mm/month during both July and August sometimes resulting in flooding. During these two months, the maximum daily rainfall recorded was 127 and 140 mm, respectively. In October and November rainfall was relatively low (24 and 66 mm, respectively). Density of intermediate host snails was high in August (Fig. 2b-d) where all habitats contained water and water level was generally high in the canals due to the requirement for irrigation water. In fish ponds, thiarid snails were most common and found throughout the study period (Fig. 2b). In August, *Bithynia fuchsiana* and *Stenothyra messageri* were found in small water canals and in rice fields with *B. fuchsiana* dominating (Fig. 2c and 1d). Cercarial infections in snails were common in August (Fig. 2b-d). Shortly after transplanting the rice (either July/August or March), farmers were often observed to collect apple snails (*Pomacea* spp.) from rice fields to protect the young rice plants.

#### The winter period (December-January)

There was a slight decline in prevalence and density of infection in fish from November to January (Fig. 2a) and this coincided with a lowering of the water level in canals in preparation for the rice harvest (early December) and the lower temperatures (Fig.1c) during the winter months (monthly average air temperature was 18.4 °C and 16.6 °C in December and January, respectively). During the December sampling for snails, all rice fields were dry and had been ploughed. Rice fields lay fallow until February when they were prepared for rice planting in March. Some farmers, however, grew vegetables in small plots in the rice fields during the winter season. The water level in most small canals was generally low in January and some were dry. Snail density was high in canals and many snails were infected (Fig. 2c), although this could partly be due to sampling efficiency being higher when the water level is low and only dissection was used to detect infections. Some ponds had high water level depending on water level in surrounding canals, while others had lower levels. Density of the thiarid snails was still high in December and infections were prevalent (Fig. 2b-d).

In mid-January, sites in rice fields were still dry and the water level in small canals was generally low; this coincided with a decline observed in the density of the thiarid snails and prevalence of infection in canals (Fig. 2c). Many farmers removed bottom sediment from their fish ponds and spread it within their compound for germination of the rice seeds. The removal of mud from fish ponds was done after lowering the water level and this activity removed a considerable number of snails although this was not reflected in the estimated snail density (Fig. 2b). During the sampling (around January 20) canals were rapidly filled and the dry rice fields were flooded. No snails, however, could be found in the newly flooded fields.

#### The spring period (February-May)

Prevalence and especially density of FZT infection in fish was lower in March (not significant) than in December but with the increase in temperature from March trematode transmission seemed to increase (Fig. 2a). The snail sampling in March was just after rice had been transplanted and the water level was generally high in all habitats. Farmers were observed to use pesticides in the rice fields. *Bithynia fuchsiana* was the dominating species in rice fields while in small canals, thiarid snails and *Stenothyra messageri* were the dominating species (Fig. 2c).

#### Associations between FZT infections in fish and snail density

Since we did not have detailed information about activities related to the management in agriculture (water management due to rice culture) and aquaculture (emptying of ponds or mud removal, possible selective removal of fish species and others), it was decided to test the effect of snail occurrence only during the period of intense transmission from July to November. For this analysis, snail density estimated from the sampling in August was used as predictor of infection in pond fish, but since host snails were found in relatively few sites in rice fields and small canals, snail counts were recoded as absent or present. For ponds, host snail counts greater than zero were split into two groups (low and high density) with the same number of ponds; ponds without snails constituted the reference group. The results of this analysis are shown in Table 6.

**Table 6:** Statistical analysis of metacercariae counts and FZT infection status (not infected, infected) of fish collected in 25 ponds located in two communes, Nhgia Lac and Nhgia Phu, in Nam Dinh province, northern Vietnam during the main FZT transmission season (July-November)

Factor (No. of sites sampled)	No. of fish	Intensity of infection		Infection status	
		Count ratio (uni-variable^a^)	Count ratio (multi-variable)	Odds ratio (uni-variable^a^)	Odds ratio (multi-variable)
Time of sampling					
August (25)	254	1.00		1.00	
October (22)	221	1.47	1.90*	1.88**	1.98**
December (21)	213	2.77*	2.98***	2.84***	3.22***
Fish species (ponds)					
*C. idellus* (20)	91	1.00	1.00	1.00	1.00
*H. molitrix* (21)	192	0.80		0.63	
*C. mrigala* (21)	90	0.07***	0.14*	0.52	
Other species (19)	119	0.21**		0.26*	0.34***
*L. rohita* (25)	196	0.04***	0.04***	0.20***	
Commune					
Nghia Lac (12)	365	1.00		1.00	1.00
Nghian Phu (13)	323	1.80		2.41***	2.29***
Snail density					
August in ponds					
None (5)	119	1.00	1.00	1.00	
Low (10)	293	1.29		1.01	
High (10)	276	2.26	2.62*	1.34	
August in small canals					
Absent (19)	525	1.00		1.00	
Present (6)	163	0.61		0.74	
August in rice fields					
Absent (17)	462	1.00		1.00	
Present (8)	226	0.65		1.17	

^a^Univariable tests are not really uni-variable because time of sampling was included as well; * p < 0.05; ** p < 0.01; *** p < 0.001

Density of host snails (no snails, low and high density) in ponds was not significantly associated with density of infection in fish when tested by univariable analysis (Table 6). In the multivariable analysis, however, high snail density was significantly associated with higher metacercariae density in fish (Table 6). In this model both time of sampling and fish species were significant predictors of metacercariae density as well (Table 6). *Ctenopharyngodon idellus, Hypophthalmichthys* and *Cirrhinus mrigala* did not differ significantly while *Labeo rohita* and other fish species had lower metacercariae density than the first three species combined (Table 6). Prevalence of infection showed the same pattern (Table 6). Density of snails releasing pleurolophocercous cercariae did not provide significant prediction of infections in fish. The number of ponds from where snails were found positive for pleurolophocercous cercariae during the individual sampling rounds ranged from 4 to 6 ponds only, and for all sampling rounds combined, infected (pleurolophocercous cercariae only) snails were recorded at least once from 13 ponds. The number of sites with infected snails in small canals ranged from 1 to 5 in the individual rounds; infected snails were recorded at least once from a total of 9 sites in small canals. Similar figures from rice fields were 1–3 (excluding rounds where rice fields were dry), and infected snails were recorded at least once at four sites.

Similarly, host snails were recorded in 8 to 20 ponds during the six surveys, and host snails were recorded at least once in 23/25 ponds. For small canals, host snails were recorded in 4 to 10 sites, with infected snails being recorded at least once from 14 sites. During individual samplings, host snails were found in 3 to 8 sites in rice fields and from 13 sites host snails were recorded at least on one occasion.

## Discussion

Our results show that host snails and FZT infections were found in all three types of habitat surveyed, i.e. rice fields, small canals and ponds, and that infection in cultured fish to some extent was related to density of potential intermediate host snails within the ponds. This was previously observed also in nursery ponds in Nam Dinh Province [14] and in the River Mekong Delta [27] but in both areas prevalence and intensity of infections in fish could be high even when snail population density within ponds was low and possibly nil. Although we showed that FZT cercariae are produced in snails especially from small canals, the presence of snails in small canals or rice fields did not significantly predict infection level in pond fish. Unless a fish pond is constructed within the perimeter of a rice field, there is probably little exchange of water between rice fields and ponds although this may happen during periods of flooding which occur regularly. Many ponds, however, are connected to a small canal through a screened tube and water can pass in both directions depending on where water level is highest. Although FZT infected snails were found in relatively few sites in small canals in our study, snail infection levels in small canals was high in some sites; often, however, infection levels are low and finding infected snails may not happen unless sampling effort is greatly increased. Furthermore, cercariae might be transported some distance with water flow in these small canals and hence not necessarily produced at the water intake for ponds.

Our results showed that FZT transmission to fish was intense from April to November and subsided thereafter due to the low temperatures and possibly the reduced water level in the area. During the winter period many parts of canals and rice fields dry out as irrigation stops. Snails found during the winter months, however, were infected but cercariae emergence during this period was not detected. During winter the low temperature arrests or slows down the cercariae release from snails. Temperature is important for trematode transmission and temperature changes as a result of climate change might result in changed transmission patterns or intensity of transmission [27–31]. Experimental studies show that infected snails kept at low temperature may temporarily produce more cercariae when transferred to a higher temperature [32]. Similar findings were described for transmission of *Schistosoma haematobium* in Madagascar, i.e. during the cold months snails could still become infected and infections then matured in synchrony with increased temperature resulting in very intense transmission [33]. Whether the same could happen in the case of FZT transmission in Nam Dinh remains to be studied.

Some grow-out ponds are emptied during the winter period and mud removed from the bottom is spread in nearby gardens or fields as fertilizer but some may be used for germination of rice seeds, which are then later transferred to nurseries or fields. Mud removal will also remove a substantial part of the snail population depending on how it is done [13, 34].

Transmission of FZT in the fish ponds studied resumed after the winter period in late February/March and ponds that were emptied during winter would be restocked in March/April. In addition, other ponds may have their fish stock supplemented with juvenile fish. During the winter period fish harvest could result in reduced overall infection levels depending on the species harvested, i.e. selective harvesting of either *Ctenopharyngodon idellus* (Grass carp) or *Labeo rohita* (Indian carp) would reduce and increase respectively overall prevalence of FZT infection in the pond. Alternatively, there could also be a die-off of established metacercariae in the fish flesh, but metacercariae seems to survive for a long time in carp [35].

Variability in snail population density among sites was pronounced due to the specific agricultural or aquaculture practices at a given location. Obviously, rice fields undergo the most pronounced seasonal changes due to field preparation, transplanting of rice and harvesting. Shortly after fields were flooded in preparation for the next rice transplanting, *Bithynia* spp. (primarily *B. fuchsiana*), *Lymnaea viridis* and small planorbid snails were found at high density and not surprisingly we found the snail fauna in rice fields related to that in the surrounding small canals although some species seem to prefer rice fields over small canals (*Lymnaea viridis* and *Bithynia fuchsiana*). Especially during the transplanting of rice seedlings and for some time thereafter, the density of *Bithynia* spp. was found to be very high. *Parafossarulus striatulus*, an important intermediate host for *Clonorchis sinensis* [7], is not as common as *Bithynia fuchsiana* and we did not record the former species at our study sites, but it can be found in rice fields as well and occasionally at very high density (unpublished data).

In small canals snail density and diversity can be high but can be affected by activities such as duck keeping, although that is more common in large canals. Vegetation removal may be done as part of canal maintenance or for use as feed for *C. idellus*. Small canals are also an important habitat and source for some small-sized fish species or juveniles of the cultured species. This natural occurring fish stock is exploited by farmers and fish is caught mainly with small seine nets. Thus small canals could contribute significantly to FZT infections in the final hosts (humans, dogs and cats).

In ponds, the presence of snails is influenced by activities such as introducing aquatic plants from other habitats as food for Grass carp [16] and snails collected from other habitats and added as food for *Mylopharyngodon piceus*, which primarily feed on snails [21]or for ducks [20]. Other activities we observed that facilitate FZT transmission to snails is cleaning of slaughtered animals in ponds, especially intestines of dogs and cats, as these are important final hosts of both intestinal and liver trematodes.

The trematode fauna in our study area was quite diverse with 12 morphotypes, some of which might represent more trematode species and besides the FZT types other species are potentially zoonotic, such as the echinostomes [20, 36] and *Fasciola* spp. [37]. Echinostome cercariae were commonly found in all habitats and have ducks and chickens as the main final hosts [20] which are infected when eating snails or fish containing echinostome metacercariae. Ducks are often kept in pens in ponds or canals, but some may also forage freely in the rice fields. Some echinostomes may also be zoonotic [36]. Fasciolosis is prevalent in the Red River Delta [38–40] but although we examined about 1800 *Lymnaea viridis*, primarily collected in rice fields, we did not detect cercariae that could be referred to *Fasciola* spp. Most likely rice fields are not contaminated with eggs of *Fasciola* spp. as cattle are not allowed into the rice fields but farmers use cattle manure on the field before planting rice.

## Conclusion

In conclusion, FZT transmission to fish in the study area was intense from June to November but then slowed down during the winter months (December-January) due to low temperatures and lower level of agricultural activities. Cercariae emergence from snails seems to stop or was significantly reduced during the winter months. Transmission of FZT within ponds to some extent was related to density of the intermediate host snails and although our results did not determine the importance of especially small canals, these habitats probably are important as they exchange water with ponds. FZT cercariae were produced in snails from all three habitats studied. Control of these infections can only be achieved through a holistic approach [13] including measures to reduce egg contamination of the environment (treatment of infections in the final hosts, sanitary measures, prevention of animals’ access to the pond and changed management practices) and reduction of the intermediate snail host density in the ponds and surrounding habitats.
